# Supplementary material for: Model Identifies Genetic Predisposition of Alzheimer’s Disease as Key Decider in Cell Susceptibility to Stress
Source: Int J Mol Sci. 2021 Nov 5;22(21):12001. doi: 10.3390/ijms222112001 (PMC8584528; doi:10.3390/ijms222112001)
Supplement: Supplementary file 1 [file ijms-22-12001-s001.zip › ijms-1424941-supplementary.pdf]

Supplementary Materials

Model Identifies Genetic Predisposition of Alzheimer's Disease as Key Decider in Cell Susceptibility to Stress

1. Primer Sequences

Table S1. Sequences of primers used for quantitative real-time PCR (qRT-PCR)

Gene	GenBank accession No.	Forward Primer (5'-3')	Reverse Primer (5'-3')	Amplicon size (bp)
APP	NM_201414.2	GGCCCTGGAGAACTACATCA	AATCACACGGAGGTGTGTCA	199
ATF4	NM_001675.2	TCAAACCTCATGGGTTCTCC	TGTCATCCAACGTGGTCAG	226
BiP/GRP78	NM_005347.4	GCGGGTGGCAGCGACAGAGCC	GCCCGGGCTGGGGAATGACCACT	474
CHOP	NM_004083	CTCTGGCTTGGCTGACTGA	GCTCTGGGAGGTGCTTGT	62
XBP-1 total	NM_001079539.1	AGGCCCAGTTGTCACCCCTCC	CCCAGCTCCGGAACGAGGTCA	441
XBP-1 spliced	NM_005080.3	CCGCAGCAGGTGCAGG	GAGTCAATACCGCCAGAATCCA	70
18S rRNA	M10098	GTAACCCGTTGAACCCCATT	CCATCCAATCGGTAGTAGCG	171

2. Computational Model

Model development has followed a bottom-up approach. We have formulated a kinetic model that describes known features of the IRE1α, PERK and ATF6 pathways. The complete map of reactions and signals considered in the model is shown in Figure S1.

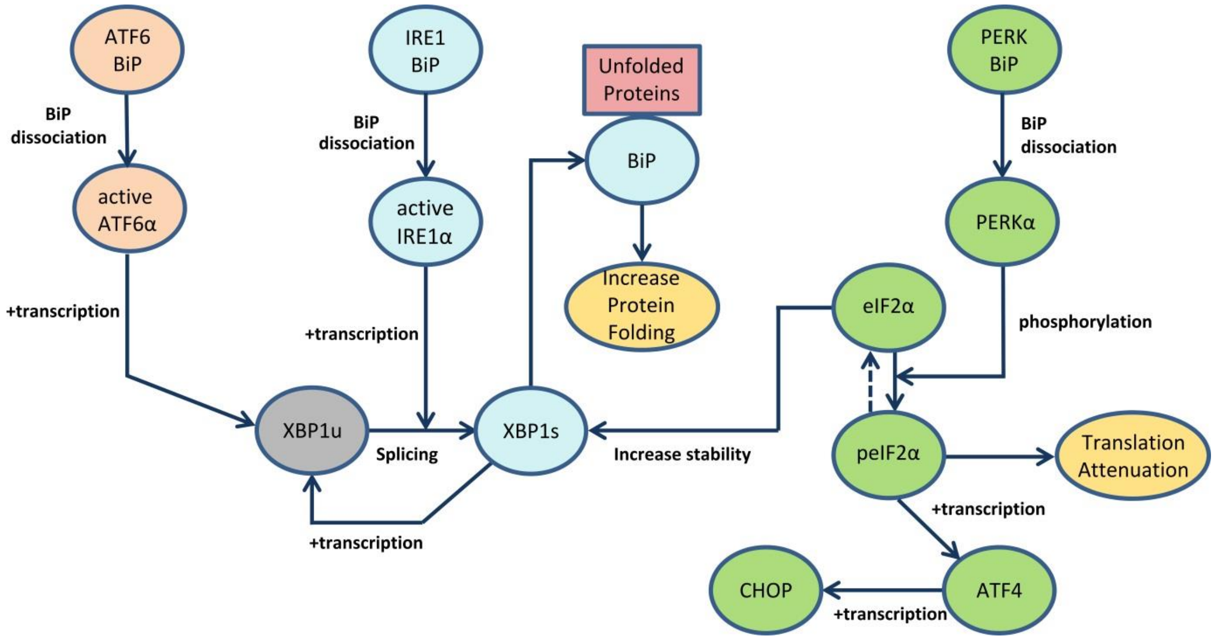

Figure S1: The mammalian UPR network

Activation and deactivation of the ER receptors and thus of UPR is modulated by BiP. More specifically, its dissociation from the receptors induces their activation while its binding induces the deactivation of the stress response. Activation of the IRE1 pathway was studied by probing the XBP1 components, activation of the PERK pathway by probing the phosphorylated eIF2α and the downstream ATF4 and CHOP genes while activation of the ATF6 pathway by probing XBP1u.

2.1 Nomenclature

$ATF4$	ATF4 protein (molecules)
$ATF4_m$	ATF4 mRNA (molecules)
$ATF6_A$	ATF6 protein (molecules)
B_m	BiP mRNA (molecules)
C_{AB}	Binding rate of BiP to ATF6 receptor (molecule^{-1} s^{-1})
C_{IB}	Binding rate of BiP to IRE1α receptor (molecule^{-1} s^{-1})
C_{PB}	Binding rate of BiP to PERK receptor (molecule^{-1} s^{-1})
C_{RB}	Binding rate of BiP to ATF6 or/and IRE1α or/and PERK (molecule^{-1} s^{-1})
C_{UB}	Binding rate of BiP to unfolded proteins (molecule^{-1} s^{-1})
$CHOP$	CHOP protein (molecule)
$CHOP_m$	CHOP mRNA (molecule)
$eIF2a$	eIF2a protein (molecule)

IRE	IRE1 protein (molecule)
IRE_A	Activated IRE1 protein (molecule)
IRE/B	IRE1-BiP complex (molecule)
K_U	Unfolded protein load (molecule s^{-2})
n	Constant for decay rate of activated IRE1 (dimensionless)
N_{ATF4_m}	Constant for transcription rate of ATF4 mRNA under UPR (dimensionless)
N_{Bm}	Constant for transcription rate of BiP mRNA under UPR (dimensionless)
N_{CHOP_m}	Constant for transcription rate of CHOP mRNA under UPR (dimensionless)
$N_{ATFF\alpha}$	Constant for magnitude of stress response following ATF6 activation (dimensionless)
$peIF2a$	Phosphorylated eIF2a protein (molecule)
$PERK_A$	PERK protein (molecule)
$PERK/B$	PERK-BiP complex (molecule)
S_U	Rate of unfolded protein translation (molecule s^{-1})
t	Time (s)
U	Unfolded protein (molecule)
U/B	Folding complex (molecule)
X_{Total}	Total XBP1 mRNA (molecule)
$X^s{}_m$	Spliced XPB1 mRNA (molecule)
$X^u{}_m$	Unspliced XPB1 mRNA (molecule)

Greek symbols

α_0	Constant for transcription rate of BiP, ATF4 and CHOP mRNA (dimensionless)
α_1	Constant for transcription rate of BiP, ATF4 and CHOP mRNA (dimensionless)
β_{ATF4}	Translation rate of ATF4 mRNA (s^{-1})
β_{ATF4_m}	Transcription rate of ATF4 (molecule s^{-1})
β_{ATF6}	Formation rate of ATF6 (molecule s^{-1})
β_B	Translation rate of BiP mRNA (s^{-1})
β_{Bm}	Basal transcription rate BiP mRNA (molecule s^{-1})
β_{CHOP}	Translation rate of CHOP protein (s^{-1})
β_{CHOPm}	Basal transcription rate of CHOP mRNA (molecule s^{-1})
$\beta_{eIF2\alpha}$	Phosphorylation rate of eIF2α (s^{-1})
$\beta_{X_m^s}$	Splicing rate of XPB1 mRNA (s^{-1})
$\beta_{X_m^u}$	Basal transcription rate of unspliced XBP1 mRNA (molecule s^{-1})
γ_{ATF4}	Decay rate of ATF4 protein (s^{-1})
γ_{ATF4_m}	Decay rate of ATF4 mRNA (s^{-1})
γ_B	Decay rate of BiP protein (s^{-1})
γ_{Bm}	Decay rate of BiP mRNA (s^{-1})
γ_{CHOP}	Decay rate of CHOP protein (s^{-1})
γ_{CHOP_m}	Decay rate of CHOP mRNA (s^{-1})
$\gamma_{eIF2\alpha}$	eIF2α dephosphorylation rate (s^{-1})
γ_{fold}	Protein folding rate (s^{-1})
γ_{AB}	Dissociation rate of BiP from ATF6 (s^{-1})
γ_{IB}	Dissociation rate of BiP from IRE1 (s^{-1})
γ_{PB}	Dissociation rate of BiP from PERK (s^{-1})

γ_{RB}	Dissociation rate of BiP from ATF6 or/and IRE1α or/and PERK (s^{-1})
γ_s	Decay rate of spliced XBP1 mRNA (s^{-1})
γ_u	Decay rate of unspliced XBP1 mRNA (s^{-1})
γ_{UB}	Dissociation rate of BiP from unfolded protein (s^{-1})
$\gamma_{X_m^s}$	Modified decay rate of spliced XBP1 mRNA (s^{-1})

2.2 Model Development

The development of the computational model is based on the following assumptions:

1. Activation of IRE, PERK and ATF6 occurs from the dissociation of BiP from these receptors.
2. The binding and dissociation constants between BiP and the three receptors take the same values for all pathways. The values are based on the model for the IRE1 pathway in yeast by Pincus *et al.* [1].
3. The number of IRE1 molecules in the ER was chosen to be the same as in the yeast system [1]. The number of PERK and ATF6 molecules in the ER was chosen to be the same as the number of IRE1 molecules. All three receptors were assumed to be bound to BiP initially.
4. The translation rate of BiP mRNA, β_B ,is the same as in yeast which is 0.25 s^{-1}[2].
5. Decay rate of BiP protein, γ_B , is 1.39×10^{-4} s^{-1} [3].
6. The initial values for *ATF4, CHOP, eIF2a, U, U|B* were chosen arbitrarily such that the initial state of the single-cell model reflects non-stressed conditions.

2.2.1 Protein Folding Dnamics

With regards to protein folding dynamics, several key features must be taken into account in order to capture translation attenuation:

- All cell lines exhibited a delay in the onset of ER stress following Tm addition.
- Proteins upregulated due to UPR activation, i.e. chaperones, ATF4 and CHOP, are not affected by translational attenuation[4].
- Translational attenuation is dependent on phosphorylation of eIF2α[5].

We have created a progressive onset of stress, which mimics the accumulation of unfolded proteins in the ER. Therefore, the rate of unfolded proteins synthesised in the ER, S_u, is given by the following equation:

$$\frac{d[S_u]}{dt} = K_u t \qquad (1)$$

Where K_u represents the cumulative load of unfolded proteins and t is time. We have followed the approach proposed by Trusina *et al.* in their model of translation attenuation[6]. To model translation attenuation, S_u is scaled according to the extent of eIF2α phosphorylation:

$$\frac{d[U]}{dt} = \frac{[eIF2\alpha]}{[eIF2\alpha]_{(0)}} S_U - C_{UB}[U][B] + \gamma_{UB}[U|B] + \gamma_B[U|B] \qquad (2)$$

Where $[U]$ represents the concentration of unfolded proteins, $[eIF2\alpha]$ stands for eIF2a concentration, C_{UB} is the binding rate constant of unfolded protein binding to BiP, $[B]$ is the concentration of BiP, γ_{UB} is dissociation rate of BiP from unfolded protein, γ_B is the decay rate of BiP protein, and $[U|B]$ is the concentration of unfolded proteins bound to BiP. Similarly, the material balance for the unfolded protein-BiP complex is:

$$\frac{d[U|B]}{dt} = C_{UB}[U][B] - \gamma_{UB}[U|B] - \gamma_B[U|B] - \gamma_{fold}[U|B] \quad (3)$$

Where γ_{fold} is the rate of protein folding.

The rate of change of BiP mRNA is given by:

$$\frac{d[B_m]}{dt} = \beta_{Bm} + N_{Bm} f(X_m^s) - \gamma_{Bm}[B_m] \quad (4)$$

With the transcription Hill function for the UPRE promoter:

$$f(X_m^s) = \frac{[X_m^s]^2}{\alpha_0 + \alpha_1[X_m^s] + [X_m^s]^2} \quad (5)$$

Where $[B_m]$ is the concentration of BiP mRNA, β_{Bm} is its basal transcription rate, N_{Bm} is a constant for the upregulation of BiP transcription under the UPR, and γ_{Bm} is the decay rate of BiP mRNA. $[X_m^s]$ is the concentration of spliced XBP1 mRNA and α_0, α_1 are constants. Similarly, the material balance for BiP is:

$$\frac{d[B]}{dt} = \beta_B[B_m] - \gamma_B[B] - C_{RB}[U][B] + \gamma_{RB}[U|B] + \gamma_{fold}[U|B] \quad (6)$$

Where $[B]$ is the concentration of BiP, β_B is the translation rate of BiP mRNA, C_{RB} is the rate of BiP binding to the free receptor, and γ_{RB} is the rate of dissociation of BiP from the inactive receptor.

2.2.2 IRE1α Pathway

The description of the IRE1 pathway follows the model proposed by Pincus *et al.*[2] for UPR activation in yeast. This consists of a single-stranded response mediated by IRE1α. It has been proposed that in the mammalian UPR system IRE1 may be activated by dissociation from BiP or by subsequent interaction of its peptide-binding domain on its luminal ER side with unfolded proteins[7,8]. These scenarios represent the single and double activation mechanism, respectively. Simulation results (data not shown) show little difference between the two activation mechanisms in terms of the overall progression of the stress pathway. For this reason, and to limit over-parameterisation, the single activation mechanism was employed.

Under non-stressed conditions, IRE1 is present in a complex with BiP ($IRE|B$) the material balance for which is:

$$\frac{d[IRE|B]}{dt} = C_{IB}[B][IRE_A] - \gamma_{IB}[IRE|B] \quad (7)$$

Where $[IRE_A]$ is the concentration of activated IRE1, C_{IB} is the binding rate of BiP to activated IRE1, and γ_{IB} is the dissociation rate of BiP from IRE1. Activated IRE1 is described by:

$$\frac{d[IRE_A]}{dt} = -C_{IB}[B][IRE_A] + \gamma_{IB}[IRE|B] \tag{8}$$

The material balance for the unspliced XBP1 mRNA (X_m^u) is described as a Hill function:

$$\frac{d[X_m^u]}{dt} = \beta_{X_m^u} + N_{ATF6\alpha} f(ATF6_\alpha) \tag{9}$$

Where $\beta_{X_m^u}$ the basal transcription rate of unspliced XBP1 mRNA, $N_{ATF6\alpha}$ is the constant representing the magnitude of stress response following ATF6 activation, and $f(ATF6_\alpha)$ is the following activation Hill function:

$$f(ATF6_A) = \frac{[ATF6_A]^2}{\alpha_0 + \alpha_1[ATF6_A] + [ATF6_A]^2} \tag{10}$$

Where $[ATF6_A]$ is the concentration of activated ATF6 and α_0, α_1 are constants.

The material balance for XPB1 spliced mRNA is:

$$\frac{d[X_m^s]}{dt} = -\gamma_{X_m^s}[X_m^s] + \beta_{X_m^s} min([IRE_A], [X_m^u]) \tag{11}$$

Spliced XBP1 mRNA stability is dependent on the level of phosphorylation of eIF2α. To capture this feature, we introduced a modified decay rate of spliced XBP1 mRNA that is linearly dependent on the concentration of phosphorylated eIF2α. Our approach was that at 0% of phosphorylation, the decay rate for the mRNA would be the same as the one of the unspliced form taken from Majumder *et al* [9] . In contrast, at full phosphorylation, the decay rate would be the one of the spliced form as given in the same study. The decay rate of sXBP1 would therefore become:

$$\gamma_{X_m^s} = \gamma_u + (\gamma_s - \gamma_u)\frac{[peIF2\alpha]}{[eIF2\alpha]_{(0)}} \tag{12}$$

Where $\begin{cases} \gamma_u = 5.7 \times 10^{-4} s^{-1} \\ \gamma_s = 8.7 \times 10^{-5} s^{-1} \end{cases}$

This new feature also allowed us to create a link (other than BiP and unfolded proteins) between the two pathways; such links are known to be multiple[10]. Finally adding the downstream pathway of PERK with mRNAs and protein such as ATF4 and PERK also helps us create a negative regulation loop in the PERK pathway, through the CHOP-mediated dephosphorylation of phosphorylated eIF2α [11].

2.2.3 PERK Pathway

Under non-stressed conditions, PERK is bound to BiP. In the presence of increased levels of unfolded protein in the ER, BiP dissociates from this complex and PERK becomes activated. The material balances for the complex ($[PERK|B]$) and the activated PERK ($[PERK_A]$) are :

$$\frac{d[PERK|B]}{dt} = C_{PB}[B][PERK_A] - \gamma_{PB}[PERK|B] \tag{13}$$

$$\frac{d[PERK_A]}{dt} = -C_{PB}[B][PERK_A] + \gamma_{PB}[PERK|B] \quad (14)$$

Where C_{PB} is the binding rate of BiP to activated PERK and γ_{PB} is its dissociation rate from the complex.

Following on from PERK activation, eIF2α is phosphorylated. The material balances for the phosphorylated ($[peIF2\alpha]$) and non-phosphorylated ($[eIF2\alpha]$) species are as follows:

$$\frac{d[peIF2\alpha]}{dt} = \beta_{eIF2\alpha} min([PERK_A], [eIF2\alpha]) - \frac{[CHOP]}{[CHOP]_{max}} \gamma_{eIF2\alpha}[peIF2\alpha] \quad (15)$$

$$\frac{d[eIF2\alpha]}{dt} = -\beta_{eIF2\alpha} min([PERK_A], [eIF2\alpha]) + \frac{[CHOP]}{[CHOP]_{max}} \gamma_{eIF2\alpha}[peIF2\alpha] \quad (16)$$

Where $\beta_{eIF2\alpha}$ is the rate of phosphorylation and $\gamma_{eIF2\alpha}$ the rate of dephosphorylation.

Dephosphorylation of peIF2a is mediated by GADD34 [11] which is directly activated by CHOP [12]. We have therefore used the ratio of $[CHOP]/[CHOP]_{max}$ as a modulator of the dephosphorylation rate.

As part of the UPR, ATF4 is transcribed and translated. The former event is subject to regulation by the level of phosphorylated eIF2α, which is described as a Hill function in the material balance below:

$$\frac{d[ATF4_m]}{dt} = \beta_{ATF4_m} + N_{ATF4_m} f(peIF2\alpha) - \gamma_{ATF4_m}[ATF4_m] \quad (17)$$

With $f(peIF2\alpha) = \frac{[peIF2\alpha]^2}{\alpha_0 + \alpha_1[peIF2\alpha] + [peIF2\alpha]^2}$

Where β_{ATF4_m} is the basal transcription rate of ATF4, N_{ATF4_m} is a constant for the upregulation of ATF4 transcription under the UPR, and γ_{ATF4_m} is the decay rate of ATF4 mRNA.

The material balance for ATF4 protein is:

$$\frac{d[ATF4]}{dt} = \beta_{ATF4}[ATF4_m] - \gamma_{ATF4}[ATF4] \quad (18)$$

Where β_{ATF4} is the translation rate of ATF4 mRNA and γ_{ATF4} is the decay rate of ATF4 protein.

The next effect of the PERK pathway is the expression of CHOP. CHOP mRNA ($[CHOP_m]$) is given by:

$$\frac{d[CHOP_m]}{dt} = \beta_{CHOPm} + N_{CHOP_m} f(ATF4) - \gamma_{CHOP_m}[CHOP_m] \quad (19)$$

where

$$f(ATF4) = \frac{[ATF4]^2}{\alpha_0 + \alpha_1[ATF4] + [ATF4]^2} \quad (20)$$

β_{CHOPm} and γ_{CHOP_m} are the transcription and decay rates of CHOP mRNA, respectively. N_{CHOP_m} is a constant for the upregulation of CHOP transcription under the UPR.

Finally, the levels of CHOP are:

$$\frac{d[CHOP]}{dt} = \beta_{CHOP}[CHOP_m] - \gamma_{CHOP}[CHOP] \tag{21}$$

Where β_{CHOP} is the translation rate of CHOP mRNA and γ_{CHOP} is the decay rate of CHOP protein.

2.2.4 ATF6 Pathway

Under homeostatic conditions, ATF6 is bound to BiP. In the presence of increased levels of unfolded protein in the ER, BiP dissociates from this complex and ATF6 translocates to the Golgi where it gets cleaved by caspases. This results in the release of its activated truncated form. The material balances for the complex ($[ATF6|B]$) and the activated ATF6 ($[ATF6_A]$) are :

$$\frac{d[ATF6|B]}{dt} = C_{AB}[B][ATF6_A] - \gamma_{AB}[ATF6|B] \tag{22}$$

$$\frac{d[ATF6_A]}{dt} = -C_{AB}[B][ATF6_A] + \gamma_{AB}[ATF6|B] \tag{23}$$

Where C_{AB} is the binding rate of BiP to activated ATF6 and γ_{AB} is the dissociation constant of BiP from ATF6.

2.3 Model Calibration

In order to enable model simulation, most parameter values were determined based on literature values and were maintained constant for all three cell lines studies experimentally. Their values are shown in Table S2. Certain parameters were calibrated using the experimental data presented in the manuscript using the maximum likelihood formulation of the parameter estimation entity in gPROMS (PSE Ltd., U.K.). The results of the parameter estimation for each of the three cell lines are shown in Table S3. Finally, Table S4 lists the initial conditions used for model simulation.

Table S2. Parameter values obtained from literature sources

Model parameters	Value	Units	Reference
C_{AB}	2.86	$mol^{-1} s^{-1}$	1
C_{PB}	2.86	$mol^{-1} s^{-1}$	
C_{IB}	2.86	$mol^{-1} s^{-1}$	
C_{RB}	2.86	$mol^{-1} s^{-1}$	
C_{UB}	2.86	$mol^{-1} s^{-1}$	
n	4.5	AU	
S_U	0	$mol\ s^{-1}$	
β_{ATF4}	0.25	s^{-1}	
β_B	0.25	s^{-1}	
β_{CHOP}	0.25	s^{-1}	
$\beta_{eIF2\alpha}$	0.16	s^{-1}	
γ_{ATF4}	7.7×10^{-3}	s^{-1}	Value re-used from fitted γ_{CHOP}
γ_B	1.39×10^{-4}	s^{-1}	

$\gamma_{eIF2\alpha}$	8.33×10^{-4}	s^{-1}	1
γ_{AB}	196	s^{-1}	
γ_{IB}	196	s^{-1}	
γ_{PB}	196	s^{-1}	
γ_{RB}	196	s^{-1}	
γ_{UB}	196	s^{-1}	

Parameters pertaining to the kinetic rates of UPR progression were estimated from the experimental data of the APPS cells and are shown in Table S3. These were fixed for the other two cell lines, with the exception of six parameters that were estimated separately from the data set for each cell line. Parameter estimation was carried out in the gPROMS modelling environment (Process Systems Enterprise Ltd., London, U.K.) using the maximum likelihood formulation.

Table S3. Estimated parameter values

Model Parameters	WT	S	S-I	Units
α_0	181935			AU
α_1	23.73			AU
β_{ATF4_m}	0.24			mol s^{-1}
β_{Bm}	0.5×10^{-1}			mol s^{-1}
β_{CHOPm}	41.43			mol s^{-1}
$\beta_{X_m^s}$	1.42×10^{-4}			s^{-1}
$\beta_{X_m^u}$	0.9×10^{-2}			mol s^{-1}
γ_{ATF4_m}	4.67×10^{-7}			s^{-1}
γ_{Bm}	1.03×10^{-4}			s^{-1}
γ_{CHOP}	7.70×10^{-3}			s^{-1}
γ_{CHOP_m}	7.73×10^{-6}			s^{-1}
γ_{fold}	2.58×10^{-4}	6.12×10^{-4}	2.39×10^{-4}	mol s^{-1}
γ_s	3.69×10^{-8}			s^{-1}
γ_u	6.37×10^{-4}			s^{-1}
$\gamma_{X_m^s}$	3.69×10^{-8}			s^{-1}
K_u	35.10×10^3	134.28×10^3	50.04×10^3	mol s^{-2}
N_{ATF4_m}	4.89	18	4.85	AU
N_{Bm}	4	1.84	1.48	AU
N_{CHOP_m}	71.88	378.3	138.76	AU
N_{ATF6a}	0.01	0.76	0.57	AU

Table S4. Initial conditions used for model simulation

Variable	Initial condition
$ATF4$	0
$ATF4_m$	3160.39
$ATF6_A$	0
B	430000
B_m	200
$CHOP$	0
$CHOP_m$	109562.41
$eIF2a$	200
IRE_A	0
IRE/B	256
$peIF2a$	0
$PERK_A$	0
$PERK/B$	256
U	0
U/B	0
X_{Total}	151.88
X^s_m	27.54

2.4 Model Simulation Results

The model was simulated with the initial conditions and parameter values presented above and the results were compared to the experimental data, as shown in Figure S2. The agreement for the UPR target genes BiP (a-c), XBP1s (d-f) and ATF4 (g-i) for the APP_{WT} (a, d, g), APP_S (b, e, h) and APP_{S-I} (c, f, i) cell lines is satisfactory. The fitting results obtained show that the model is able to reproduce the desired shape of the UPR response for each of the three cell lines studied.

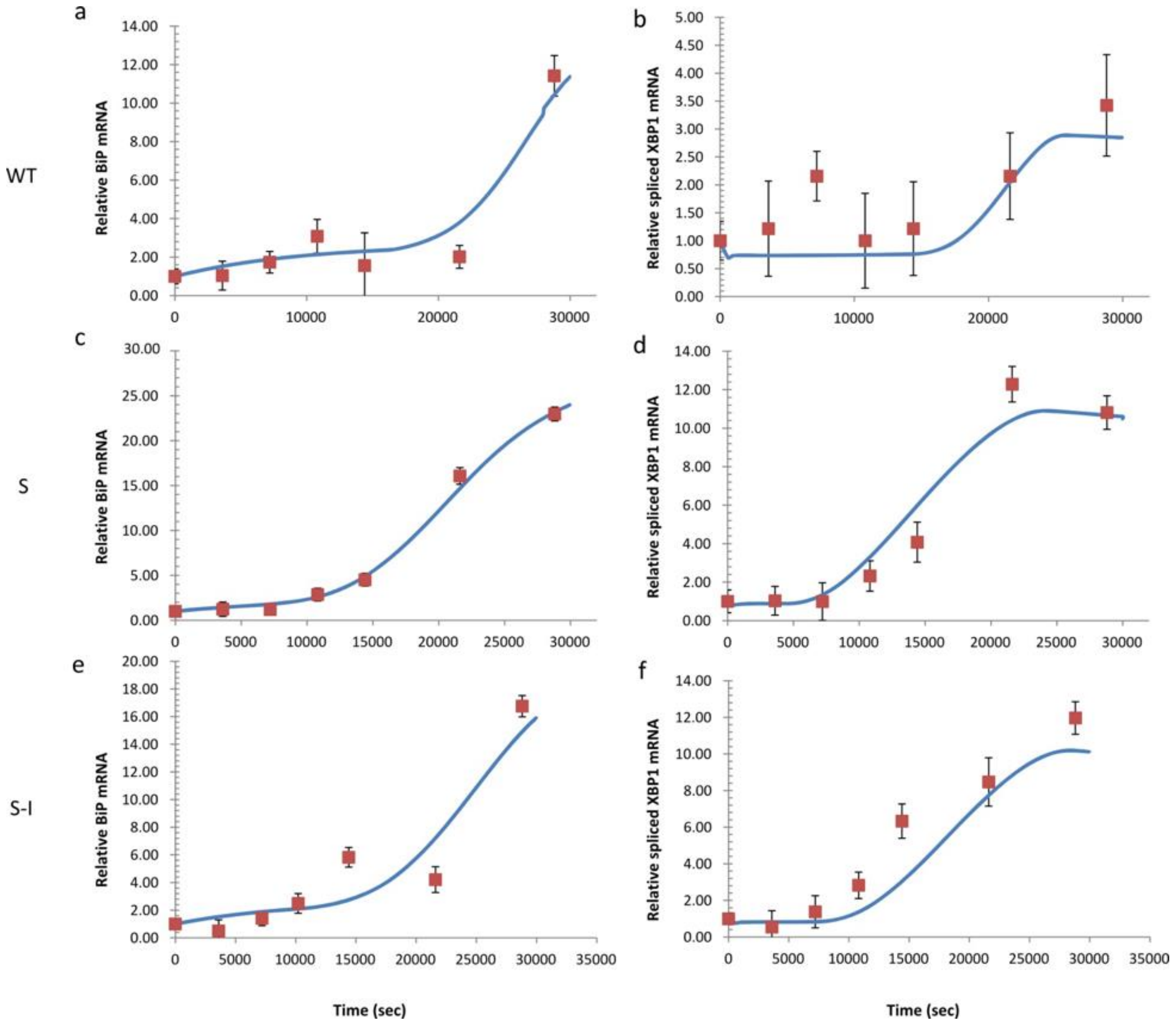

Figure S2. Comparison of model simulation results with experimental data for BiP and XBP1s mRNA

The dots correspond to the experimental data while the solid lines show the model simulation results.

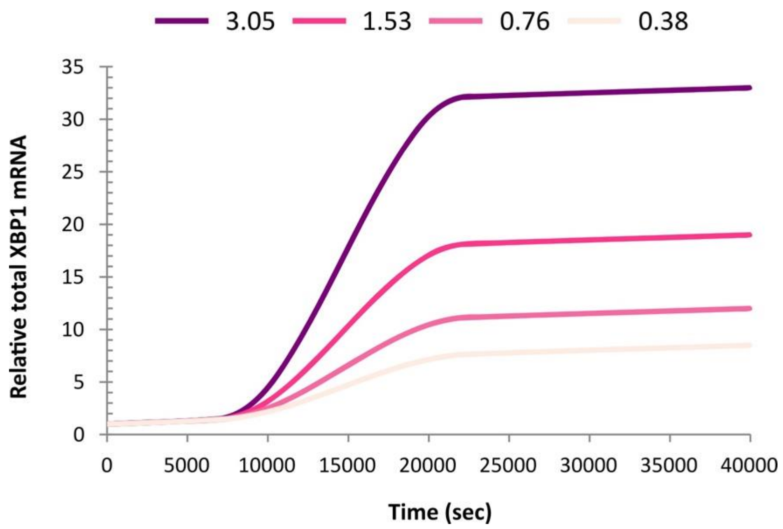

Figure S3. Sensitivity analysis of the ATF6 pathway

The plot illustrates the effect of the magnitude of stress response following ATF6 activation (N_{ATF4_A}) on the relative amount of XBP1 total mRNA levels. During stress, a higher N_{ATF4_A} causes an increased expression of the XBP1 total transcript level.

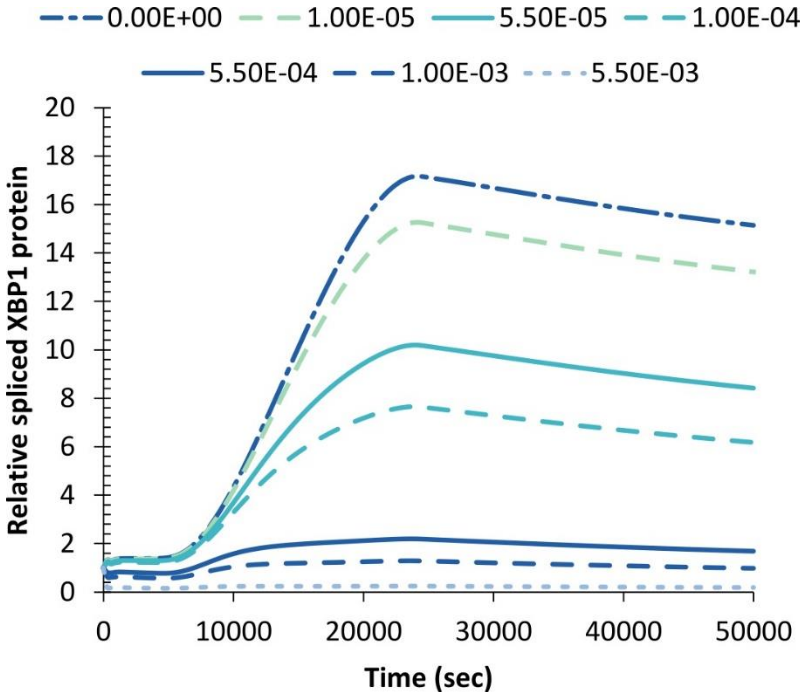

Figure S4. Sensitivity analysis of the effect of XBP1 protein complex formation rate on the XBP1s protein levels in APP_S cells during TM treatment

Spliced XBP1 protein levels are upregulated during ER stress (12,000-18,000 sec) APP_S. The pXBP1s levels are directly affected by the XBP1 protein complex formation (β_{X^c}).

References

1. Ghaemmaghami, S., W. Huh, K. Bower, R.W. Howson, A. Belle, N. Dephoure, E.K. O'Shea, and J.S. Weissman, *Global analysis of protein expression in yeast.* Nature, 2003. **425**(6959): p. 737-741.
2. Pincus, D. *et al.* BiP Binding to the ER-Stress Sensor Ire1 Tunes the Homeostatic Behavior of the Unfolded Protein Response. *PLoS. Biol.* **8** (2010).
3. Axelsen, J.B. & Sneppen, K. Quantifying the benefits of translation regulation in the unfolded protein response. *Phys Biol* **1**, 159-165 (2004).
4. Ventoso, I., Kochetov, A., Montaner, D., Dopazo, J. & Santoyo, J. Extensive Translatome Remodeling during ER Stress Response in Mammalian Cells. *Plos One* **7** (2012).
5. Hetz, C. The unfolded protein response: controlling cell fate decisions under ER stress and beyond. *Nat Rev Mol Cell Bio* **13**, 89-102 (2012).
6. Trusina, A., Papa, F.R. & Tang, C. Rationalizing translation attenuation in the network architecture of the unfolded protein response. *P Natl Acad Sci USA* **105**, 20280-20285 (2008).
7. Kimata, Y. & Kohno, K. Endoplasmic reticulum stress-sensing mechanisms in yeast and mammalian cells. *Curr Opin Cell Biol* **23**, 135-142 (2011).
8. Oikawa, D., Kitamura, A., Kinjo, M. & Iwawaki, T. Direct Association of Unfolded Proteins with Mammalian ER Stress Sensor, IRE1 beta. *Plos One* **7** (2012).
9. Majumder, M. *et al.* A Novel Feedback Loop Regulates the Response to Endoplasmic Reticulum Stress via the Cooperation of Cytoplasmic Splicing and mRNA Translation. *Mol Cell Biol* **32**, 992-1003 (2012).
10. Novoa, I., H.Q. Zeng, H.P. Harding, and D. Ron, *Feedback inhibition of the unfolded protein response by GADD34-mediated dephosphorylation of eIF2 alpha.* Journal of Cell Biology, 2001. **153**(5): p. 1011-1021.
11. Novoa, I., Zeng, H.Q., Harding, H.P. & Ron, D. Feedback inhibition of the unfolded protein response by GADD34-mediated dephosphorylation of eIF2 alpha. *J Cell Biol* **153**, 1011-1021 (2001).
12. Marciniak, S.J. *et al.* CHOP induces death by promoting protein synthesis and oxidation in the stressed endoplasmic reticulum. *Gene Dev* **18**, 3066-3077 (2004).
